# Trap-Controlled White Electroluminescence From a Single Red-Emitting Thermally Activated Delayed Fluorescence Polymer

**DOI:** 10.3389/fchem.2020.00287

**Published:** 2020-04-21

**Authors:** Yun Yang, Liuqing Yang, Xuefei Li, Lei Zhao, Shumeng Wang, Junqiao Ding, Lixiang Wang

**Affiliations:** ^1^State Key Laboratory of Polymer Physics and Chemistry, Changchun Institute of Applied Chemistry, Chinese Academy of Sciences, Changchun, China; ^2^School of Applied Chemistry and Engineering, University of Science and Technology of China, Hefei, China

**Keywords:** single white-emitting polymers, carbazole dendron, thermally activated delayed fluorescence (TADF), charge trap, dual emission

## Abstract

Single white-emitting polymers have been reported by incorporating the second-generation carbazole dendron into the side chain of a red-emitting thermally activated delayed fluorescence (TADF) polymer. Due to the prevented hole trap effect, in this case, excitons can be generated simultaneously on the polymeric host and the red TADF dopant to give a dual emission. Consequently, a bright white electroluminescence is achieved even at a dopant loading as high as 5 mol.%, revealing a maximum luminous efficiency of 16.1 cd/A (12.0 lm/W, 8.2%) and Commission Internationale de l'Eclairage (CIE) coordinates of (0.42, 0.32). The results clearly indicate that the delicate tuning of charge trap is a promising strategy to develop efficient single white-emitting polymers, whose low-band-gap chromophore content can be up to a centesimal level.

**Graphical Abstract:**
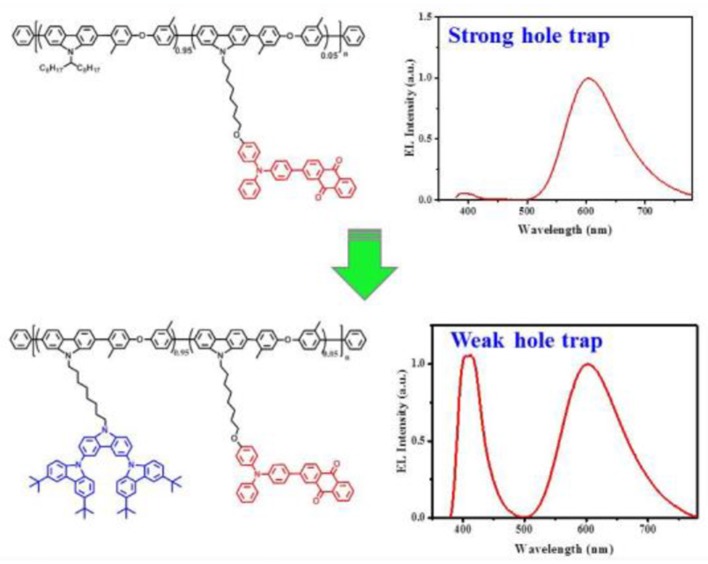
Single white-emitting polymers (SWPs) were achieved by simply introducing the second-generation carbazole dendron to suppress hole trap effect on the dopant.

## Introduction

Single white-emitting polymers (SWPs) have attracted much attention owing to their potential applications in flat-panel displays and solid-state lightings (Reineke et al., 2013). In this case, several fluorescence, phosphorescence, and/or thermally activated delayed fluorescence (TADF) chromophores with either two complementary colors (blue and yellow) or three primary colors (blue, green, and red) are covalently incorporated into a single polymeric host at the same time so as to generate white electroluminescence (EL) (Liu et al., 2007a,b; Shao et al., 2012; Li et al., 2017; Wang et al., 2017). Compared with the physical blend systems, the undesirable phase segregation can be avoided effectively, leading to improved device performance as well as good spectral stability (Tu et al., 2004). However, the molar ratio of the long-wavelength chromophores in SWPs is often required to be in the range of one ten thousandth to one thousandth (Liu et al., 2007a; Shao et al., 2012; Wang et al., 2017). The extremely low doping concentration is difficult to be controlled during polymerization, which may bring about batch-to-batch variation for the synthesis of SWPs, and thus poor device reliability and reproducibility.

**Figure 1:**
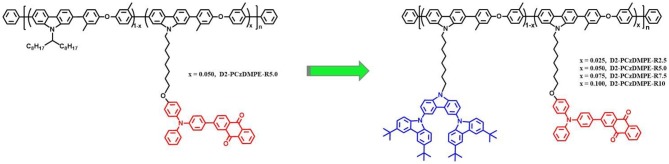
Design rule for SWPs from a red-emitting TADF polymer.

We note that there are few studies on how to address such an issue, although a great progress has been made on the power efficiency of SWPs recently (Shao et al., 2018). Here, we report TADF-based SWPs, whose low-band-gap chromophore content can be raised up to a centesimal level. This is achieved by simply introducing the second-generation carbazole dendron into the side chain of a previously-reported red-emitting TADF polymer PCzDMPE-R5.0 (Figure 1) (Yang et al., 2019). Because of the suppressed hole trap effect on the dopant, an interesting dual emission originating from both host and dopant is observed under the electrical excitation for all the resultant SWPs (D2-PCzDMPE-R2.5 ~ D2-PCzDMPE-R10). Among them, D2-PCzDMPE-R5.0 gives a more balanced white EL, revealing a maximum luminous efficiency of 16.1 cd/A (12.0 lm/W, 8.2%) and Commission Internationale de l'Eclairage (CIE) coordinates of (0.42, 0.32). The results clearly indicate that the delicate tuning of charge trap is a promising strategy to develop efficient SWPs with a high loading of long-wavelength chromophores.

## Results and Discussion

### Synthesis and Characterization

**Scheme 1:**
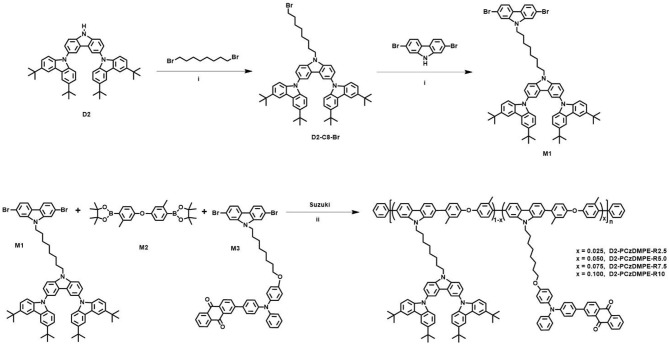
Synthetic route of TADF polymers. Reagents and conditions: (i) K_2_CO_3_, tetrabutylammonium bromid, 70°C; (ii) Pd_2_(dba)_3_, S-Phos, Aliquat 336, K_2_CO_3_, H_2_O, toluene, 96°C.

The synthetic route of the TADF-based SWPs is depicted in Scheme 1. Starting from the second-generation oligocarbazole D2, two successive N-alkylated reactions were carried out to afford the key monomer M1. Combined with other two comonomers M2 and M3, then a Suzuki polymerization was adopted to produce the target polymers D2-PCzDMPE-R2.5, D2-PCzDMPE-R5.0, D2-PCzDMPE-R7.5, and D2-PCzDMPE-R10. Their number-average molecular weights and polydispersity indexes were determined to be 63–101 kDa and 1.58–1.66, respectively (Table 1). And the actual content of the red TADF dopant incorporated into polymer can be calculated using their ^1^H NMR spectra. As one can see in Figure S1, the characteristic signals of δ8.46 and 8.28 are subjected to the anthraquinone segment in the red TADF emitter, while the peak at about δ2.31 is from the methyl group in the 3,3′-dimethyldiphenyl ether building block. By comparing their relative integrals, the red dopant loading is estimated to be in the range of 2.4–10.0%, very close to the feed ratio (Table 1). This implies that the red TADF emitter has been successfully bonded into the SWPs during polymerization.

**Table 1:** Physical properties of the polymers D2-PCzDMPE-R2.5 ~ D2-PCzDMPE-R10.

**Polymers**	**Red emitter content in the polymers (mol%)**		**M_**n**_^b^ (kDa)**	**PDI ^b^**	**T_**g**_^c^ (° C)**	**T_**d**_^d^ (° C)**
	**Feed ratio**	**Actual content^a^**				
D2-PCzDMPE-R2.5	2.5	2.4	64	1.61	266	455
D2-PCzDMPE-R5.0	5.0	5.0	63	1.58	262	470
D2-PCzDMPE-R7.5	7.5	7.4	101	1.62	264	460
D2-PCzDMPE-R10	10.0	10.0	100	1.66	259	457

a *Calculated from the ^1^H NMR spectra*.

b *Determined by GPC in THF using polystyrene as the standard*.

c *Glass transition temperatures determined by TGA in N_2_*.

d *Decomposition temperatures corresponding to a 5% weight loss*.

In addition, they all exhibit a decomposition temperature (T_d_: corresponding to a 5% weight loss) of 455–470°C and a glass transition temperature (T_g_) of 259–266°C (Figure S2), much higher than those of PCzDMPE-R5.0 (T_d_ = 417°C, T_g_ = 94°C) (Yang et al., 2019). The introduced oligocarbazole functionalized with tert-butyl groups may be responsible for the improved thermal stability of D2-PCzDMPE-R2.5 ~ D2-PCzDMPE-R10 (Zhao et al., 2015, 2018). Also, it contributes to their good solubility in common organic solvents (toluene, chlorobenzene, and chloroform etc.), which ensures the generation of high quality films via spin coating.

### Photophysical Properties

**Figure 2:**
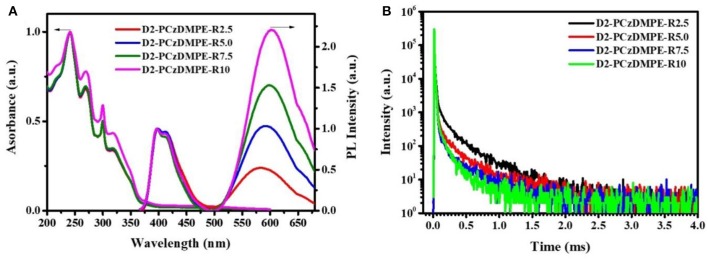
Photophysical properties of D2-PCzDMPE-R2.5 ~ D2-PCzDMPE-R10: **(A)** UV-vis absorption and PL spectra in films; **(B)** transient PL spectra in films under N_2_ at 298 K.

The UV-Vis absorption and photoluminescent (PL) spectra of D2-PCzDMPE-R2.5 ~ D2-PCzDMPE-R10 were firstly measured in neat films to investigate their photophysical properties. As can be clearly seen in Figure 2A, these SWPs show not only the absorptions peaked at 241 and 300 nm from the tethered oligocarbazole dendron, but also the elongated absorption bands of 270 and 319 nm from the backbone poly(2,7-carbazole-co-3,3′-dimethyldiphenyl ether) (Zhao et al., 2015). And the charge transfer (CT) absorption related to the red TADF dopant seems to be weak but distinguishable, lying in the range of 400–550 nm. Due to the incomplete energy transfer, moreover, a dual emission is detected for all the polymers. One is from the blue polymeric host, the other is from the red TADF dopant, whose intensity is found to be gradually increased with the increasing doping concentration.

To demonstrate their TADF nature, the film transient PL spectra were also recorded under nitrogen at 298 K for D2-PCzDMPE-R2.5 ~ D2-PCzDMPE-R10 (Figure 2B and Figure S3). Obviously, they display both the prompt fluorescence with an excited lifetime of 7.0–10.8 ns, and the delayed fluorescence with an excited lifetime of 97–157 us. When the feed ratio of the red TADF dopant rises, it is found that the delayed lifetime is down from 157 us of D2-PCzDMPE-R2.5 to 136 us of D2-PCzDMPE-R5.0, 126 us of D2-PCzDMPE-R7.5, and 97 us of D2-PCzDMPE-R10, respectively (Table 2). The trend may be ascribed to the triplet-triplet annihilation (TTA) induced by aggregation. This is further verified by their film PL quantum yields (PLQYs) (Tao et al., 2014). For example, the PLQY of D2-PCzDMPE-R10 (Φ_P_ = 0.24) is reduced by about 37% compared with D2-PCzDMPE-R2.5 (Φ_P_ = 0.38).

**Table 2:** Photophysical properties of the polymers D2-PCzDMPE-R2.5 ~ D2-PCzDMPE-R10.

**Polymers**	**λ_abs_^a^ [nm]**	**λ_em_^a^ [nm]**	**Φ_P_^b^**	**τ_p_^c^ [ns]**	**τ_d_^c^ [us]**
D2-PCzDMPE-R2.5	241/270/300/319/475	398/415/584	0.38	10.8	157
D2-PCzDMPE-R5.0	241/270/300/319/476	399/413/592	0.32	8.4	136
D2-PCzDMPE-R7.5	241/270/300/319/478	397/412/598	0.29	8.3	126
D2-PCzDMPE-R10	241/270/300/319/478	397/413/603	0.24	7.0	97
PCzDMPE-R5.0 (Yang et al., 2019)	317/492	393/579	0.37	5.9	165

a *Measured in film at 298 K*.

b Measured by integrating-sphere in film under N_2_ atmosphere for TADF polymers;

c *Measured in film N_2_ atmosphere for TADF polymers, and the prompt and delayed lifetimes (τ_p_ and τ_d_) were calculated using τ_av_ = ∑A${}_{\text{i}}\tau_{\text{i}}^{2}$/∑A_i_τ_i._*.

### Electroluminescent Properties

To evaluate the electroluminescent (EL) properties of D2-PCzDMPE-R2.5 ~ D2-PCzDMPE-R10, at first, the non-doped devices were fabricated with a configuration of ITO/PEDOT:PSS (50 nm)/EML (40 nm)/TmPyPB (50 nm)/Liq (1 nm)/Al (Figure S4). Herein PEDOT:PSS [poly(3,4-ethylenedioxythiophene): poly(styrenesulfonate)] and TmPyPB [1,3,5-tris(3-pyridyl-3-phenyl)benzene] serve as the hole-injection layer and electron-transporting layer, respectively. And the emitting layer (EML) is composed of the polymer D2-PCzDMPE-R2.5, D2-PCzDMPE-R5.0, D2-PCzDMPE-R7.5, or D2-PCzDMPE-R10 independently.

**Figure 3:**
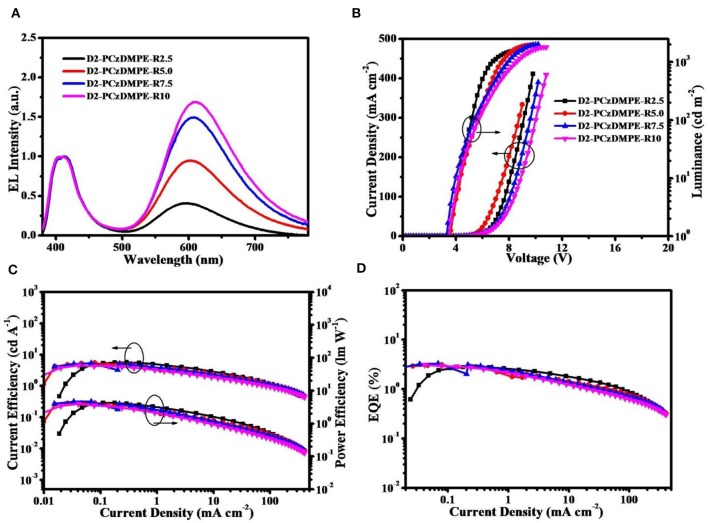
Non-doped device performance of D2-PCzDMPE-R2.5 ~ D2-PCzDMPE-R10: **(A)** EL spectra at 1,000 cd m^−2^; **(B)** current density–voltage–luminance characteristics; **(C)** current efficiency and power efficiency as a function of current density; **(D)** EQE as a function of current density.

Figure 3 plots the EL spectra, the current density-voltage-luminance characteristics together with the current efficiency, power efficiency and external quantum efficiency (EQE) as a function of current density, and the corresponding data are tabulated in Table 3. Similar to the PL counterparts, both red and blue emissions are observed in the EL spectra (Figure 3A and Figure S5). Ongoing from D2-PCzDMPE-R2.5 to D2-PCzDMPE-R5.0, D2-PCzDMPE-R7.5, and D2-PCzDMPE-R10, the red-to-blue ratio is enhanced, and the corresponding CIE coordinates are red-shifted from (0.36, 0.27) to (0.45, 0.33), (0.48, 0.36) and (0.49, 0.36), respectively. Following such a tendency, the current density at the same driving voltage is also found to be reduced (Figure 3B), indicative of the existed charge trap to some degree (Tong et al., 2007; Tsung and So, 2008; Li et al., 2012). Despite this, the turn-on voltages defined at 1 cd/m^2^ are in the range of 3.4–3.6 V for D2-PCzDMPE-R2.5 ~ D2-PCzDMPE-R10, lower than that of PCzDMPE-R5.0 (6.2 V) (Yang et al., 2019). The reduced driving voltage may originate from the introduction of oligocarbazole as the side chain, which can facilitate the hole injection and transporting (Promarak et al., 2007, 2008; Hasan et al., 2017; Wang et al., 2018). As a result, D2-PCzDMPE-R5.0 achieves the best white device performance among these polymers, revealing a maximum luminance of 1,971 cd/m^2^, a peak current efficiency of 5.4 cd/A, a peak power efficiency of 4.3 lm/W and a peak EQE of 3.1% (Figures 3C,D).

**Table 3:** Non-doped and doped device performance for the polymers D2-PCzDMPE-R2.5 ~ D2-PCzDMPE-R10.

**Devices**	**V_**on**_ (V)**	**Max performance**				**CIE (x, y)**
		***L* (cd/m^**2**^)**	**CE (cd/A)**	**PE (lm/W)**	**EQE (%)**	
D2-PCzDMPE-R2.5	3.6	1,622	5.7	4.2	2.9	(0.36, 0.27)
D2-PCzDMPE-R5.0	3.6	1,971	5.4	4.3	3.1	(0.45, 0.33)
D2-PCzDMPE-R7.5	3.4	2,008	5.3	4.7	3.3	(0.48, 0.36)
D2-PCzDMPE-R10	3.6	1,809	4.6	4.0	3.1	(0.49, 0.36)
D2-PCzDMPE-R5.0 +mCP	4.2	1,837	16.1	12.0	8.2	(0.42, 0.32)
PCzDMPE-R5.0 (Yang et al., 2019)	6.2	1,386	3.24	1.46	1.74	(0.57, 0.42)

*V_on_, turn-on voltage at 1 cd/m^2^; L, luminance; CE, current efficiency; PE, power efficiency; EQE, external quantum efficiency; CIE (x, y), CIE@1000 cd/m^2^*.

**Figure 4:**
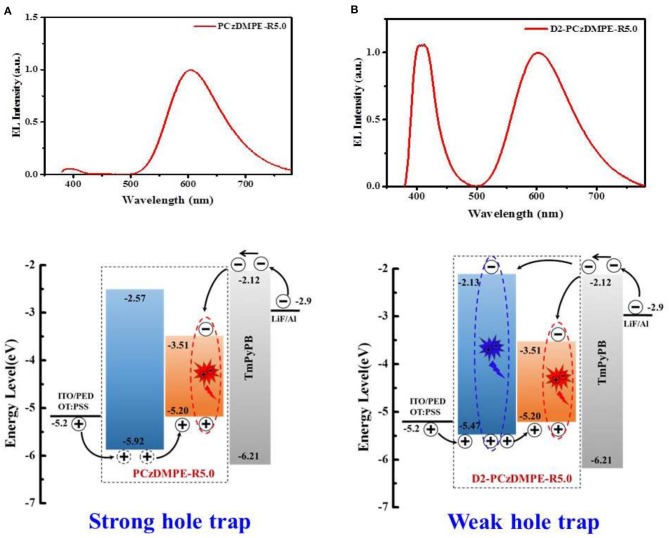
Comparison of the EL spectra and proposed working mechanism between PCzDMPE-R5.0 **(A)** and D2-PCzDMPE-R5.0 **(B)**.

Unlike the red-emitting PCzDMPE-R5.0, we note, D2-PCzDMPE-R5.0 obtains a dual emission from both the polymeric host and small-molecular TADF dopant (Figure 4). Given the same feed ratio, the additional second-generation carbazole dendron plays an important role on the observed difference. As for PCzDMPE-R5.0, there exists a strong hole trap effect owing to the much deeper highest occupied molecular orbital (HOMO) level of the polymeric host relative to the red TADF dopant (−5.92 eV vs. −5.20 eV). In this case, the injected holes cannot be stored on host but trapped by dopant completely, while electrons are injected into dopant via an electrostatic attraction (Adachi et al., 2000; Tessler et al., 2000; Lane et al., 2001; Gong et al., 2003). Then excitons are generated directly on dopant, and only red emission appears in the EL spectrum of PCzDMPE-R5.0 (Figure 4A). By contrast, the incorporated oligocarbazole has led to a distinct HOMO upshift from −5.92 to −5.47 eV for the polymeric host in D2-PCzDMPE-R5.0 (Figure S6). Benefitting from the suppressed hole trap, holes can be accumulated either on host or on dopant. After recombination with the injected electrons via an electrostatic attraction, two classes of excitons are able to be formed on both host and dopant, resulting in a dual emission and thus white EL (Figure 4B) (Liu et al., 2005; Farmer et al., 2011; Li et al., 2014).

**Figure 5:**
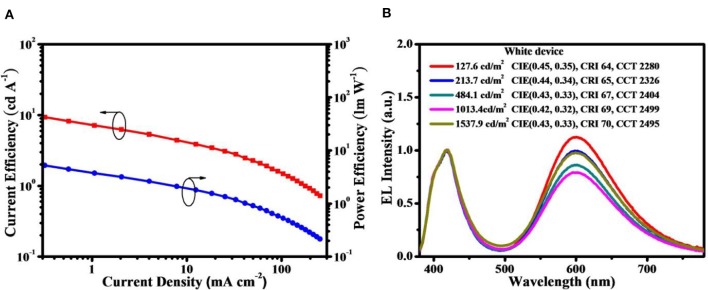
Performance for the doped device of D2-PCzDMPE-R5.0: **(A)** Current efficiency and power efficiency as a function of current density; **(B)** EL spectra at different luminance.

To avoid the above-mentioned aggregation induced TTA in neat films, doped devices were further assembled with D2-PCzDMPE-R5.0 as an example. When it is doped into 1,3-bis(9H-carbazol-9-yl)benzene (mCP) at a 30 wt.% concentration, the current efficiency, power efficiency and EQE are optimized to be 16.1 cd/A, 12.0 lm/W and 8.2%, respectively (Figure 5 and Figure S7). Meanwhile, the EL spectrum remains nearly unchanged, accompanied by similar CIE coordinates of (0.42, 0.32) to the non-doped device. Although the obtained performance is moderate, the loading of long-wavelength dopant here is as high as 5 mol.%, one or two order magnitude higher than those of previously-reported SWPs (Chuang et al., 2007; Liu et al., 2007a; Luo et al., 2007; Shao et al., 2012; Wang et al., 2017). This is very instructive when trying to solve the batch-to-batch variation in material synthesis.

## Conclusions

In summary, a red to white conversion has been demonstrated by incorporating the second-generation carbazole dendron into the side chain of a red-emitting TADF polymer. Benefitting from the elevated HOMO level of the polymeric host, the hole trap effect between host and dopant is reasonably weakened. As a consequence, a dual emission from both host and dopant is observed simultaneously, leading to a bright white EL even at a 5 mol.% dopant content. This work provides an effective strategy to improve the loading of long-wavelength chromophores up to a centesimal level, which will shed light on the development of SWPs showing not only high power efficiency but also good reproducibility.

## Experimental Section

### Measurements and Characterization

^1^H NMR and ^13^C NMR spectra were recorded with a Bruker Avance 400 spectrometer or Bruker Avance 500 spectrometer. MALDI/TOF (matrix-assisted laser desorption ionization/time-of flight) mass spectra were performed on an AXIMA CFR MS apparatus (COMPACT). Molecular weights of the polymers were determined by Gel permeation chromatography (GPC) in tetrahydrofuran (THF) using polystyrene as the standard. Thermal gravimetric analysis (TGA) and differential scanning calorimetry (DSC) were performed under a flow of nitrogen with PerkinElmer-TGA 7 and PerkinElmer-DSC7 systems, respectively. UV-vis absorption and PL spectra were measured with a PerkinElmer Lambda 35 UV-vis spectrometer and a PerkinElmer LS 50B spectrofluorometer, respectively. By dropping 0.5 ml solution of polymers dissolved in toluene to an optical colorimetric dish and then drying under vacuum, the films used for PLQY measurement are formed on the walls of the colorimetric dish. The film PLQYs were measured using a quantum yield measurement system (C10027, Hamamatsu Photonics) excited at 350 nm under argon protection. PLQYs are calculated from the area integral ratio of emission to absorption. And the transient PL spectra were carried out with Edinburgh fluorescence spectrometer (FLS980). The HOMO and lowest unoccupied molecular orbital (LUMO) levels were estimated from the cyclic voltammetry (CV), which was performed on a CHI660a electrochemical analyzer with Bu_4_NClO_4_ (0.1 mol/L) as the electrolyte at a scan rate of 100 mV/s. A glass carbon electrode, a saturated calomel electrode, and a Pt wire were used as the working electrode, the reference electrode, and the counter electrode, respectively. All the potentials were calibrated by ferrocene/ferrocenium (Fc/Fc^+^). HOMO = –e (Eox_onset_ + 4.8 V), LUMO = HOMO + E_g_, where Eox_onset_ is the onset value of the first oxidation wave and the E_g_ is the optical bandgap estimated from the absorption onset.

### Device Fabrication and Testing

The indium tin oxide (ITO) (20 Ω per square) substrates were cleaned with acetone, detergent, distilled water and then in an ultrasonic solvent bath. After baking in a heating chamber at 130°C for 2 h, the ITO-glass substrates were treated with UV-ozone for 25 min. Firstly, PEDOT:PSS (Batron-P4083, Bayer AG) was spin-coated on top of the ITO at a speed of 5,000 rpm for 60 s, and baked at 120°C for 45 min. After transferred into a nitrogen-filled glove-box, subsequently, solutions of polymers in toluene were spin-coated on PEDOT:PSS as the EML at a speed of 1,500 rpm for 60 s, and annealed at 80°C for 0.5 h. Finally, the other layers including TmPyPB (50 nm), LiF (1 nm) and Al (100 nm) were deposited in a vacuum chamber at a base pressure of >4 × 10^−4^ Pa. The EL spectra and CIE coordinates were measured using a CS2000A spectra colorimeter. The current–voltage and brightness–voltage curves of devices were measured using a Keithley 2,400/2,000 source meter and a calibrated silicon photodiode. All the measurements were carried out at room temperature under ambient conditions.

### Synthesis

All chemicals were purchased from the Energy Chemical, Aldrich, or Alfa and used without further purification unless otherwise stated. Toluene was freshly distilled before usage. Compound D2, monomer M2 and M3 were prepared according to the previous reports (Yang et al., 2018, 2019).

#### 9-(8-bromooctyl)-3,6-bis(3,6-di-tert-butyl-carbazol-9yl)-carbazole (D2-C8-Br)

A mixture of D2 (5.00 g, 6.90 mmol), 1,8-dibromooctane (9.40 g, 34.70 mmol), K_2_CO_3_ (4.80 g, 34.70 mmol), and tetrabutylammonium bromide (0.11 g, 0.35 mmol) was dissolved in 100 mL THF. The mixture was heated to reflux and stirred for 12 h under argon. After cooling to room temperature, the mixture was extracted with dichloromethane, washed with deionized water, dried by anhydrous sodium sulfate, and concentrated under vacuum. The crude product was purified by column chromatography on silica gel using petroleum ether/dichloromethane (v/v = 8/1) as eluent to give D2-C8-Br as a white powder (5.30 g, 86.5%). ^1^H NMR (400 MHz, CDCl_3_) δ 8.16 (t, J = 6.2 Hz, 6H), 7.66–7.59 (m, 4H), 7.44 (dd, J = 8.6, 1.9 Hz, 4H), 7.31 (d, J = 8.6 Hz, 4H), 4.47 (s, 2H), 3.44–3.38 (m, 2H), 2.11–1.99 (m, 2H), 1.92–1.81 (m, 2H), 1.46 (s, 44H).

#### {[3,6-bis(3,6-di-tert-butylcarbazol-9-yl)-carbazole]-9yl}octyl-2,7-dibromo-9H-carbazole (M1)

A mixture of D2-C8-Br (5.20 g, 5.69 mmol), 2,7-dibromo-9H-carbazole (1.68 g, 5.17 mmol), K_2_CO_3_ (3.57 g, 0.26 mmol) and tetrabutylammonium bromide (0.08 g, 0.35 mmol) was dissolved in 100 mL THF. The mixture was heated to reflux and stirred for 20 h under argon. After cooling to room temperature, the mixture was extracted with dichloromethane, washed with deionized water, dried by anhydrous sodium sulfate, and concentrated under vacuum. The crude product was purified by column chromatography on silica gel using petroleum ether/ethyl acetate (v/v = 20/1) as eluent. Finally, the product was recrystallized with ethyl acetate/dichloromethane to give M1 as a white solid (4.78 g, 80%), (Figure S8). ^1^H NMR (400 MHz, CDCl_3_) δ 8.16 (t, J = 4.9 Hz, 6H), 7.86 (d, J = 8.3 Hz, 2H), 7.65 – 7.57 (m, 4H), 7.53 (d, J = 1.5 Hz, 2H), 7.44 (dd, J = 8.6, 1.7 Hz, 4H), 7.35–7.27 (m, 6H), 4.44 (s, 2H), 4.21 (t, J = 7.2 Hz, 2H), 2.04–1.94 (m, 2H), 1.92–1.83 (m, 2H), 1.50–1.38 (m, 44H). MALDI-TOF MS (m/z): Calcd for C_72_H_76_Br_2_N_4_, Exact Mass: 1154.44, Found: 1154.4 (M^+^).

General synthesis of white-emitting TADF polymers with *D2-PCzDMPE-R2.5* as an example (Figure S10). M1 (0.3385 g, 0.2925 mmol), M2 (0.1351 g, 0.3000 mmol), M3 (0.0068 g, 0.0075 mmol), Pd_2_(dba)_3_ (1.1000 mg, 0.0010 mmol), 2-dicyclohexylphosphino-2,6′-dimethoxybiphenyl (S-Phos) (3.7000 mg, 0.0075 mmol) and Aliquat 336 (0.1 mL) were added to a mixture of toluene (9 mL) and aqueous K_2_CO_3_ (3.0 mL, 2 M) under argon. The mixture was heated to 95°C and stirred for 2.50 h. Subsequently, benzeneboronic acid (36 mg) in toluene of 4.5 mL was added, and the mixture was refluxed for 5 h. Then bromobenzene of 0.5 mL was added, and the mixture was kept refluxed for another 5 h. Finally, sodium diethyldithiocarbamate trihydrate (1.0 g) dissolved in deionized water (15 mL) was added into the mixture. The solution was kept at 80°C with vigorous stirring under argon for 24 h. After cooling to room temperature, the mixture was extracted by dichloromethane, which was washed for five times with deionized water and dried by anhydrous sodium sulfate. The polymer was purified by column chromatography on silica gel using dichloromethane as eluent. After removal of the solvent, the polymers were obtained by precipitation in methanol. The final purification was performed by Soxhlet extraction with acetone for about 24 h and then precipitated in methanol to give the desired polymer D2-PCzDMPE-R2.5 (251 mg, 70%). ^1^H NMR (500 MHz, CDCl_3_) δ 8.46 (s, 0.019H), 8.28 (d, J = 7.9 Hz, 0.082H), 8.23–7.94 (m, 7.481H), 7.90 (d, J = 8.1 Hz, 0.040H), 7.78–7.71 (m, 0.072H), 7.59 (dd, J = 8.6, 1.8 Hz, 2H), 7.54 (d, J = 8.6 Hz, 2H), 7.39 (m, 4H), 7.37–7.30 (m, 4H), 7.30 (s, 3H), 7.25 (s, 1H), 7.19 (t, J = 9.6 Hz, 2H), 7.06 (m, 2H), 6.98 (d, J = 8.3 Hz, 2H), 6.81 (d, J = 8.3 Hz, 0.077H), 4.34 (m, 4H), 3.87 (t, J = 6.3 Hz, 0.051H), 2.31 (s, 6H), 1.92 (m, 4H), 1.75–1.67 (m, 0.104H), 1.47–1.33 (m, 43.258H). ^13^C NMR (126 MHz, CDCl_3_) δ 156.56, 142.65, 141.00, 140.44, 140.09, 139.22, 138.24, 137.66, 131.57, 130.03, 125.89, 123.72, 123.56, 123.25, 121.63, 120.98, 120.72, 120.06, 119.63, 43.81, 43.31, 34.91, 32.25, 29.56, 29.36, 29.28, 27.63, 27.54, 21.11. Anal. calcd for [(C_86_H_88_N_4_O)_97.5_(C_66_H_54_N_2_O_4_)_2.5_]_n_: C, 86.41; H, 7.34; N, 4.66; found: C, 86.23; H, 7.45; N, 4.45.

#### D2-PCzDMPE-R5.0 (239 mg, 67%)

M1 (0.3298 g, 0.2850 mmol), M2 (0.1351 g, 0.3000 mmol), and M3 (0.0135 g, 0.0150 mmol) were used (Figure S11). ^1^H NMR (500 MHz, CDCl_3_) δ 8.46 (s, 0.038H), 8.28 (d, J = 7.8 Hz, 0.163H), 8.24–7.96 (m, 7.253H), 7.90 (d, J = 7.9 Hz, 0.084H), 7.79–7.69 (m, 0.149H), 7.59 (m, 2H), 7.54 (m, 2H), 7.40 (m, 4H), 7.33 (d, J = 7.7 Hz, 4H), 7.28 (d, J = 5.5 Hz, 3H), 7.25 (s, 1H), 7.18 (d, J = 7.9 Hz, 2H), 7.02 (d, J = 14.6 Hz, 2H), 7.01 (s, 2H), 6.81 (d, J = 8.8 Hz, 0.147H), 4.34 (m, 4H), 3.87 (t, J = 6.3 Hz, 0.106H), 2.31 (s, 6H), 2.05–1.77 (m, 4H), 1.71 (s, 0.219H), 1.54 – 1.23 (m, 42.080H). ^13^C NMR (126 MHz, CDCl_3_) δ 156.56, 142.65, 141.00, 140.44, 140.09, 139.22, 138.24, 137.66, 134.28, 134.08, 133.93, 131.57, 130.03, 129.51, 128.31, 128.09, 127.97, 127.39, 127.34, 125.89, 124.30, 123.72, 123.56, 123.25, 121.63, 120.99, 120.72, 120.06, 119.63, 116.36, 115.67, 110.07, 109.68, 109.32, 43.81, 43.31, 34.91, 32.25, 29.56, 29.36, 29.28, 27.63, 27.54, 21.11, 0.22. Anal. calcd for [(C_86_H_88_N_4_O)_95_(C_66_H_54_N_2_O_4_)_5_]_n_: C, 86.37; H, 7.31; N, 4.47; found: C, 86.30; H, 7.39; N, 4.47.

#### D2-PCzDMPE-R7.5 (212 mg, 65%)

M1 (0.3211 g, 0.2775 mmol), M2 (0.1351 g, 0.3000 mmol) and M3 (0.0203 g, 0.0225 mmol) were used (Figure S12). ^1^H NMR (500 MHz, CDCl_3_) δ 8.46 (s, 0.067H), 8.28 (d, J = 8.0 Hz, 0.230H), 8.21–7.96 (m, 7.228H), 7.90 (dd, J = 8.2, 1.8 Hz, 0.098H), 7.78–7.70 (m, 0.176H), 7.58 (dt, J = 8.1, 4.1 Hz, 2H), 7.56–7.50 (m, 2H), 7.40 (dd, J = 8.7, 1.6 Hz, 4H), 7.33 (d, J = 7.7 Hz, 4H), 7.27 (d, J = 8.3 Hz, 3H), 7.25 (s, 1H), 7.19 (t, J = 9.9 Hz, 2H), 7.10–7.02 (m, 2H), 6.98 (d, J = 8.4 Hz, 2H), 6.81 (d, J = 8.9 Hz, 0.168H), 4.34 (m, 4H), 3.87 (t, J = 6.3 Hz, 0.159H), 2.31(s, 6H), 1.99–1.83 (m, 4H), 1.76–1.65 (m, 0.200H), 1.39 (m, 41.726H). ^13^C NMR (126 MHz, CDCl_3_) δ 156.56, 142.65, 141.00, 140.44, 140.09, 139.22, 138.24, 137.66, 134.28, 134.09, 133.82, 131.57, 130.03, 129.51, 128.26, 128.09, 127.97, 127.39, 127.34, 125.89, 124.30, 123.72, 123.57, 123.25, 121.63, 120.99, 120.72, 120.06, 119.63, 116.36, 115.67, 110.07, 109.68, 109.32, 43.81, 43.30, 34.91, 32.25, 29.56, 29.36, 29.28, 27.63, 27.54, 21.11, 0.22. Anal. calcd for [(C_86_H_88_N_4_O)_92.5_(C_66_H_54_N_2_O_4_)_7.5_]_n_: C, 86.33; H, 7.27; N, 4.59; found: C, 86.00; H, 7.41; N, 4.37.

#### D2-PCzDMPE-R10 (253 mg, 72%)

M1 (0.3125 g, 0.2700 mmol), M2 (0.1351 g, 0.3000 mmol) and M3 (0.0271 g, 0.0300 mmol) were used (Figure S13). ^1^H NMR (500 MHz, CDCl_3_) δ 8.46 (s, 0.090H), 8.28 (d, J = 7.8 Hz, 0.309H), 8.22–7.95 (m, 7.166H), 7.90 (d, J = 8.1 Hz, 0.123H), 7.79–7.69 (m, 0.232H), 7.59 (dd, J = 8.6, 1.7 Hz, 2H), 7.54 (dd, J = 8.4, 3.8 Hz, 2H), 7.40 (dd, J = 8.7, 1.6 Hz, 4H), 7.33 (d, J = 7.7 Hz, 4H), 7.27 (d, J = 6.2 Hz, 3H), 7.25 (s, 1H), 7.18 (d, J = 7.9 Hz, 2H), 7.06 (m, 2H), 6.98 (d, J = 8.4 Hz, 2H), 6.81 (d, J = 8.8 Hz, 0.198H), 4.34 (m, 4H), 3.87 (d, J = 5.9 Hz, 0.206H), 2.31(s, 6H), 1.98 – 1.84 (m, 4H), 1.69 (m, 0.280H), 1.39 (m, 40.815H). ^13^C NMR (126 MHz, CDCl_3_) δ 156.56, 142.65, 141.00, 140.44, 140.09, 139.22, 138.24, 137.66, 134.29, 134.08, 133.93, 131.58, 130.03, 129.50, 128.24, 128.09, 127.97, 127.39, 127.34, 125.89, 124.31, 123.72, 123.56, 123.25, 121.63, 120.99, 120.72, 120.06, 119.63, 116.36, 115.67, 110.07, 109.68, 109.32, 43.81, 43.31, 34.91, 32.25, 29.56, 29.36, 29.28, 27.63, 27.54, 21.11, 0.22. Anal. calcd for [(C_86_H_88_N_4_O)_90_(C_66_H_54_N_2_O_4_)_10_]_n_: C, 86.28; H, 7.24; N, 4.55; found: C, 86.15; H, 7.42; N, 4.38.

#### D2-PCzDMPE (196 mg, 70%)

M1 (0.2893 g, 0.25 mmol) and M2 (0.1125 g, 0.25 mmol) were used (Figure S9). ^1^H NMR (500 MHz, CDCl_3_) δ 8.12 (m, 8H), 7.59 (dd, J = 8.6, 1.9 Hz, 2H), 7.54 (d, J = 8.7 Hz, 2H), 7.40 (dd, J = 8.7, 1.8 Hz, 4H), 7.35 (m, 4H), 7.29–7.24 (m, 4H), 7.18 (d, J = 8.1 Hz, 2H), 7.03 (d, J = 1.6 Hz, 2H), 6.98 (d, J = 9.6 Hz, 2H), 4.34 (m, 4H), 2.31(s, 6H), 1.92 (m, 4H), 1.49–1.35 (m, 44H). ^13^C NMR (126 MHz, CDCl_3_) δ 156.56, 142.65, 141.00, 140.44, 140.09, 139.22, 138.24, 137.66, 131.57, 130.03, 125.89, 123.72, 123.56, 123.25, 121.63, 120.99, 120.72, 120.06, 119.63, 116.36, 110.07, 109.68, 109.32, 43.80, 43.31, 34.91, 32.25, 29.56, 29.36, 29.28, 27.62, 27.54, 21.11. Anal. calcd for [C_86_H_88_N_4_O]_n_: C, 86.45; H, 7.37; N, 4.69; found: C, 86.45; H, 7.44; N, 4.55.

## Data Availability Statement

All datasets generated for this study are included in the article/Supplementary Material.

## Author Contributions

YY and LZ synthesized and characterized the polymers. LY and XL prepared non-doped and doped devices. SW, JD, and LW contributed conception and design of the study. YY wrote the first draft of the manuscript. JD revised manuscript. All authors contributed to manuscript revision, read, and approved the submitted version.

## Conflict of Interest

The authors declare that the research was conducted in the absence of any commercial or financial relationships that could be construed as a potential conflict of interest.

## Supplementary Material

The Supporting Information includes the ^1^H NMR and MALDI-TOF MS of M1, the ^1^H NMR and ^13^C NMR spectra, TGA curves, DSC curves, transient decay spectra of polymers, the structure of the OLED devices and molecular structures of the related materials, the molecular structures, cyclic voltammograms and energy level alignment of PCzDMPE and D2-PCzDMPE as hosts and ROC8 as guest, the current–voltage–luminance and current efficiency–luminance–power efficiency curves of white device performance.
