# Supplementary material for: Trap-Controlled White Electroluminescence From a Single Red-Emitting Thermally Activated Delayed Fluorescence Polymer
Source: Front Chem. 2020 Apr 21;8:287. doi: 10.3389/fchem.2020.00287 (PMC7186499; doi:10.3389/fchem.2020.00287)
Supplement: Supplementary file 1 [file Table_1.DOCX]

Supplementary Information (ESI)

**Trap-Controlled White Electroluminescence from a Single Red-Emitting Thermally Activated Delayed Fluorescence Polymer**

**Yun Yang^1, 2†^, Liuqing Yang^1,2†^, Xuefei Li ^1,2^, Lei Zhao^1^, Shumeng Wang^1*^, Junqiao Ding^1, 2*^, Lixiang Wang^1,2^**

^1^State Key Laboratory of Polymer Physics and Chemistry, Changchun Institute of Applied Chemistry, Chinese Academy of Sciences, Changchun, 130022, P. R. China

^2^University of Science and Technology of China, Hefei 230026, P. R. China

†These authors contribute equally to this article.

*** Correspondence:**Shumeng Wang: [wangshumeng@ciac.ac.cn](mailto:wangshumeng@ciac.ac.cn)

Junqiao Ding: [junqiaod@ciac.ac.cn](mailto:junqiaod@ciac.ac.cn)


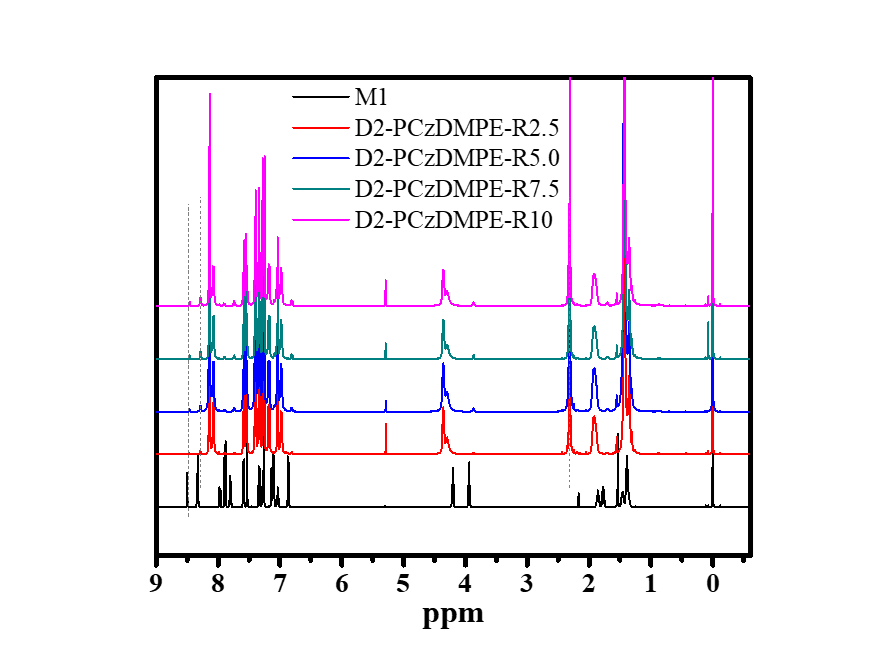


**Figure S1.** ^1^H NMR spectra of TADF polymers D2-PCzDMPE-R2.5 ~ D2-PCzDMPE-R10 compared with M1.


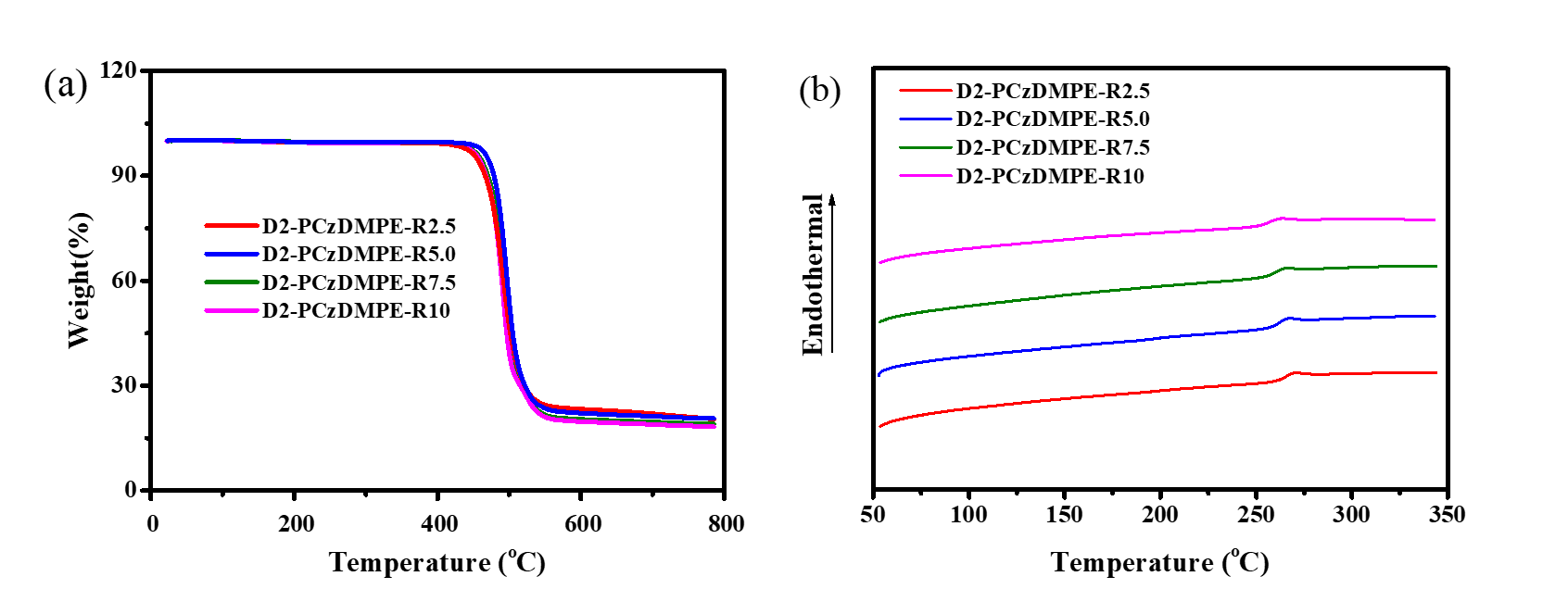


**Figure S2.** TGA (a) and DSC (b) curves of D2-PCzDMPE–R2.5、 D2-PCzDMPE–R5.0、 D2-PCzDMPE–R7.5 and D2-PCzDMPE–R10.


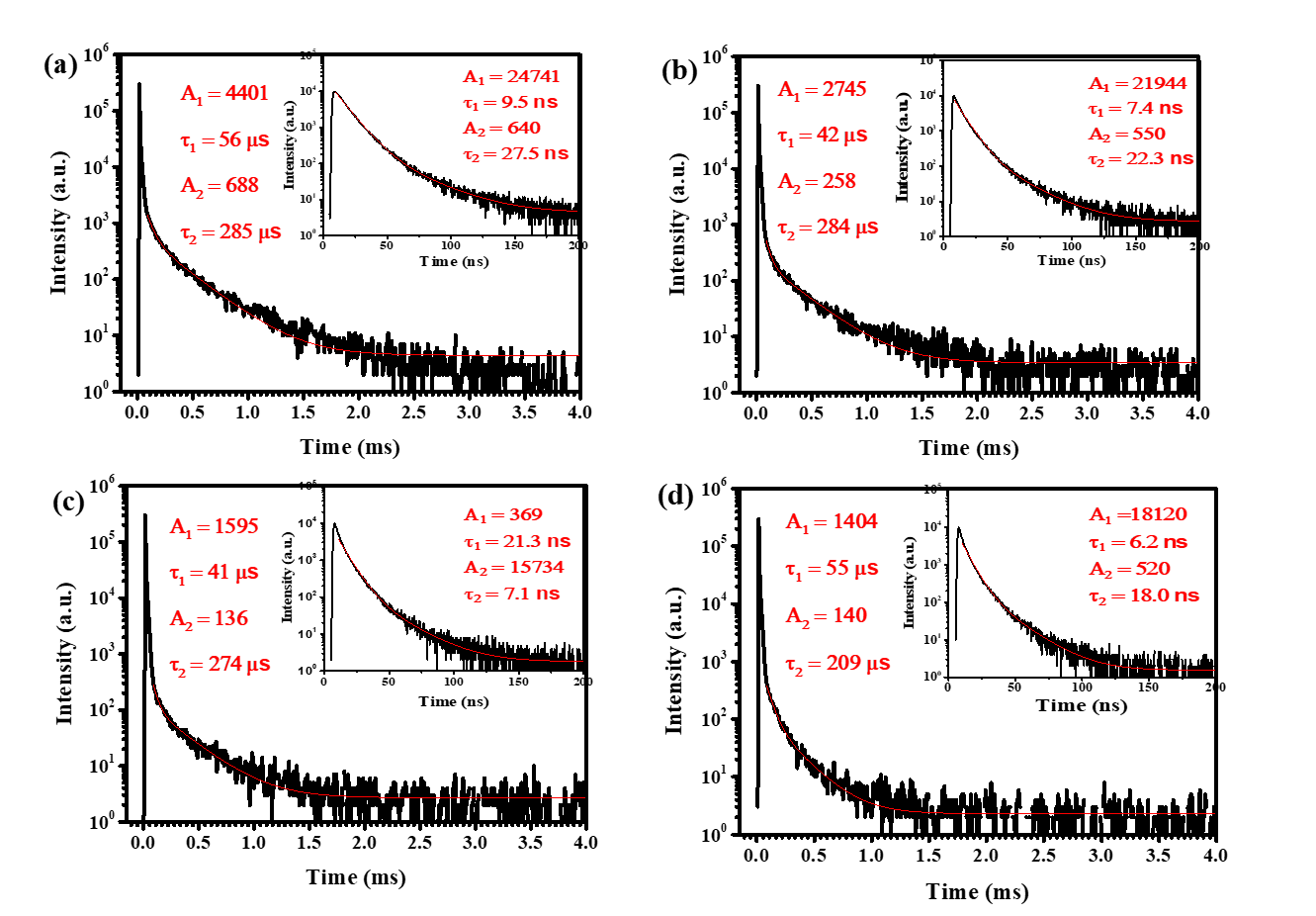


**Figure S3.** Transient decay spectra in films for D2-PCzDMPE-R2.5 (a), D2-PCzDMPE-R5.0 (b), D2-PCzDMPE-R7.5 (c) and D2-PCzDMPE-R10 (d).


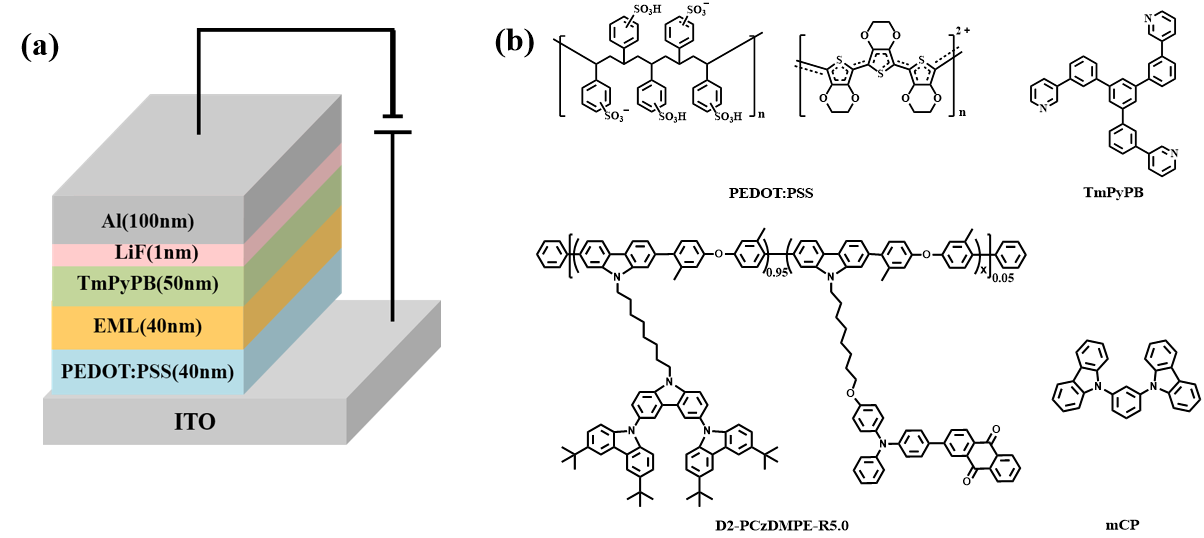


**Figure S4.** Structure of the devices (a) and molecular structures of the related materials (b).


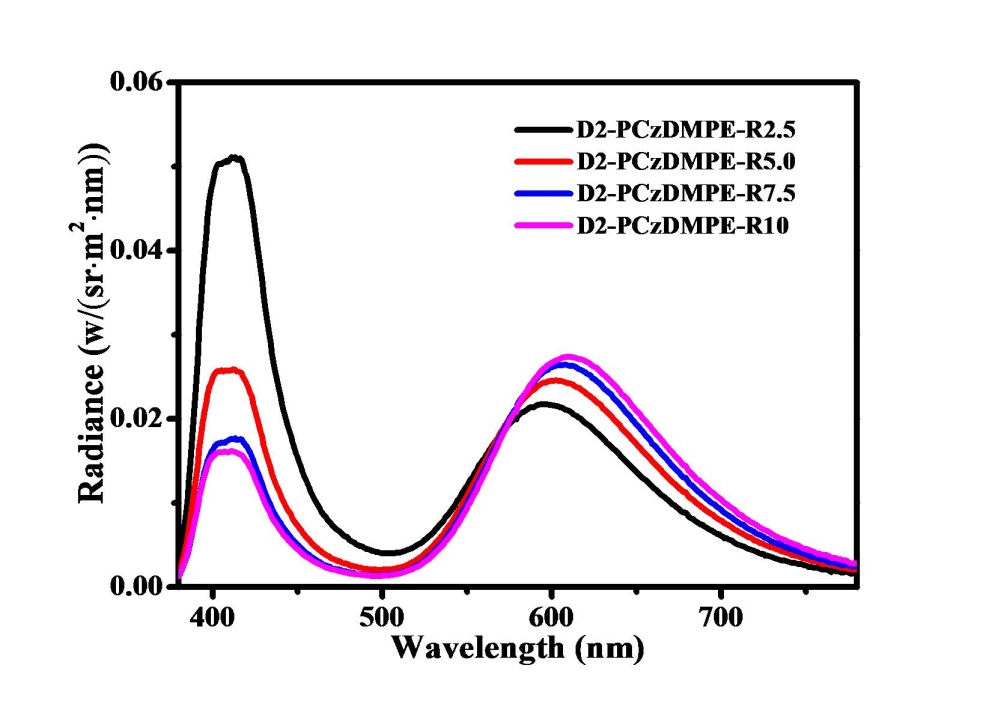


**Figure S5**. EL spectra at 1000 cd m^-2^ without normalization for nondoped devices of D2-PCzDMPE-R2.5 ~ D2-PCzDMPE-R10.


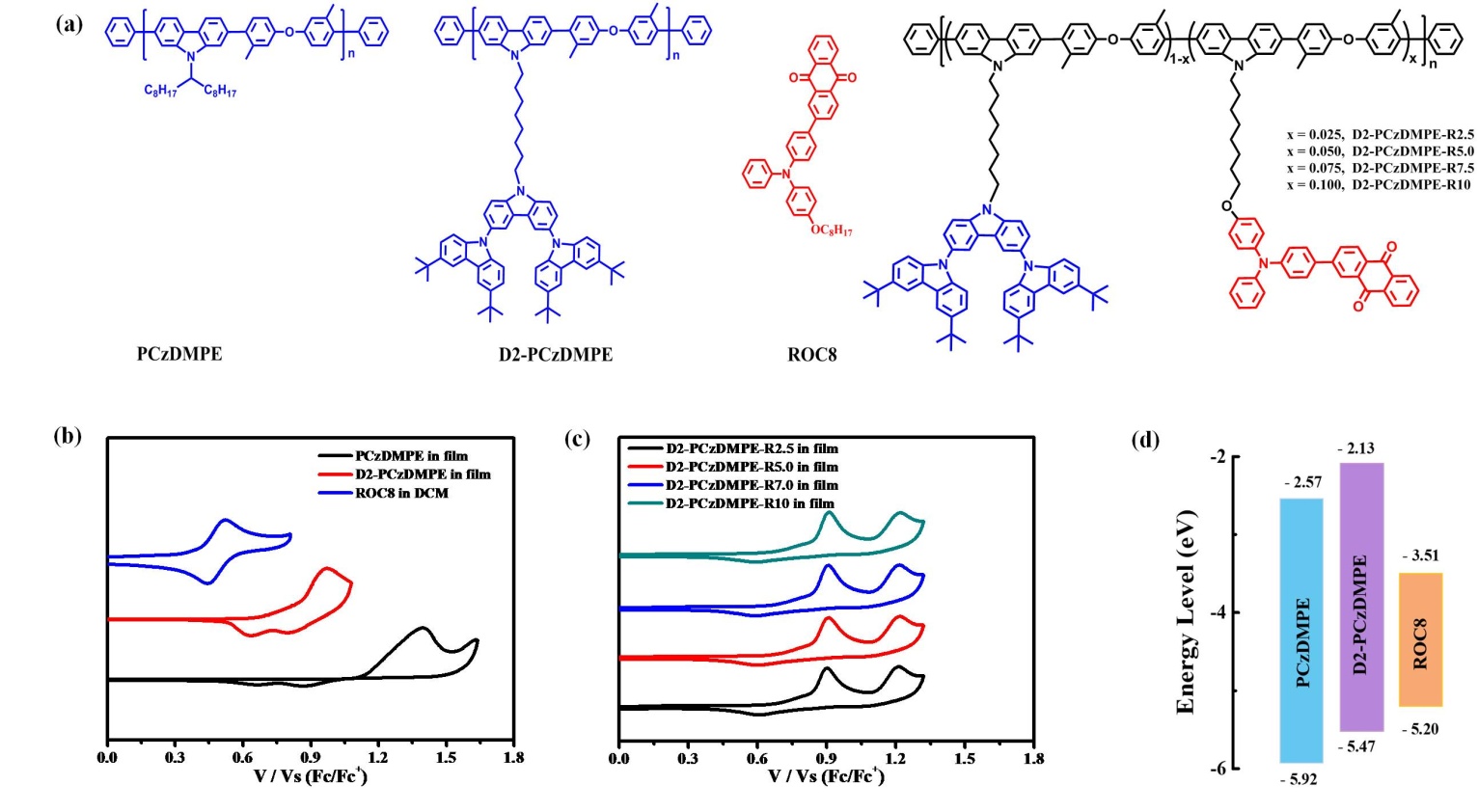


**Figure S6**. Basic properties of PCzDMPE and D2-PCzDMPE as hosts and ROC8 as guest: (a) Molecular structures; (b) and (c) Cyclic voltammograms (oxidation); (d) Energy level alignment.

**
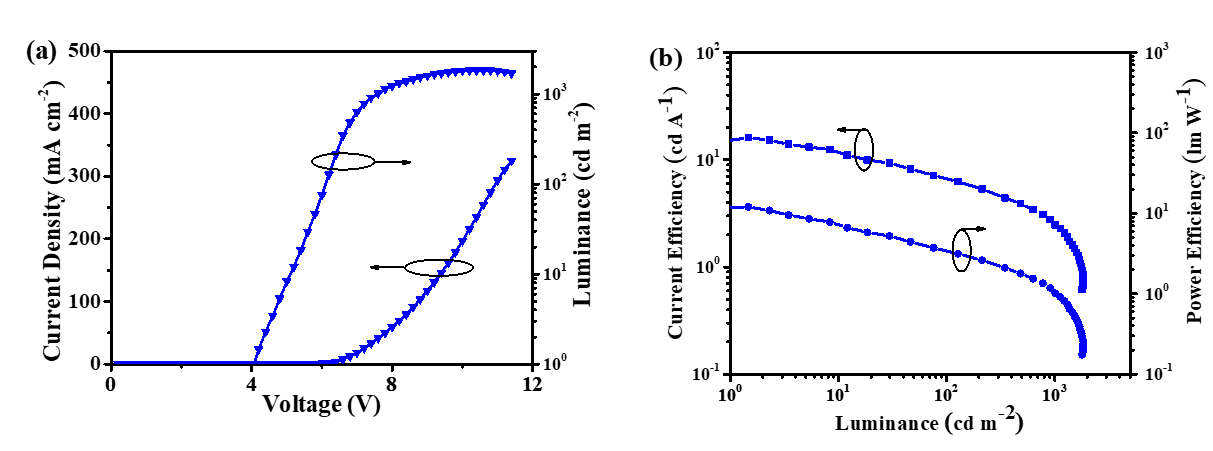
**

**Figure S7.** The current-voltage-luminance (a) and current efficiency-luminance-power efficiency (b) curves of white device performance.


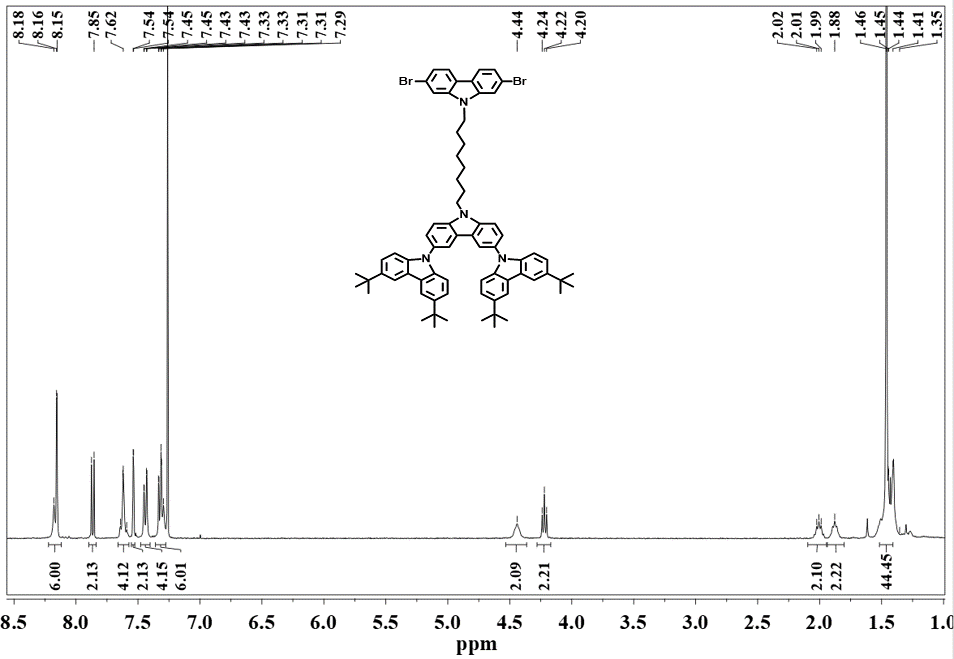


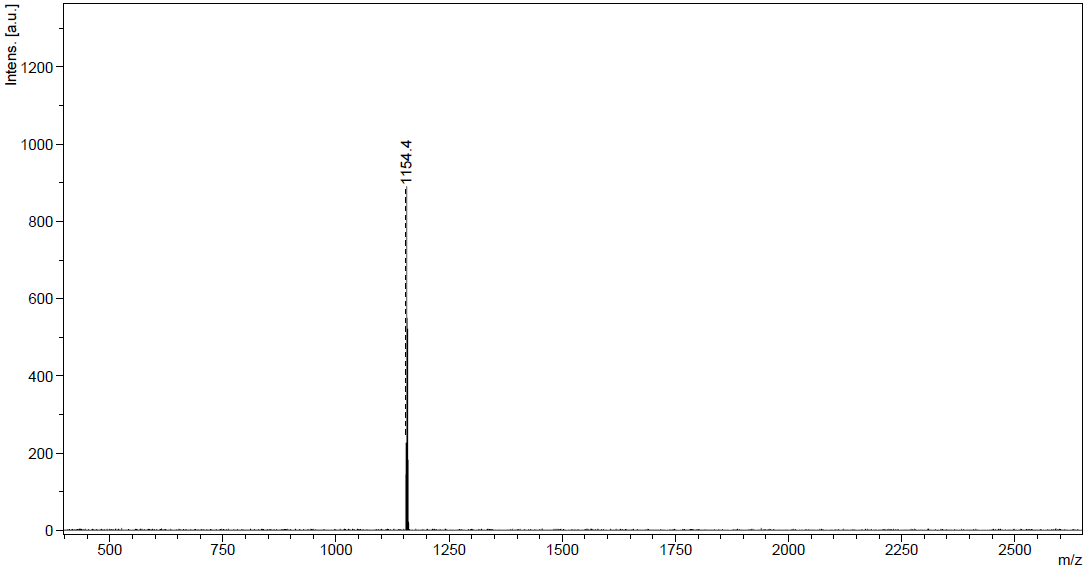


**Figure S8.** ^1^H NMR and MALDI-TOF MS of M1.


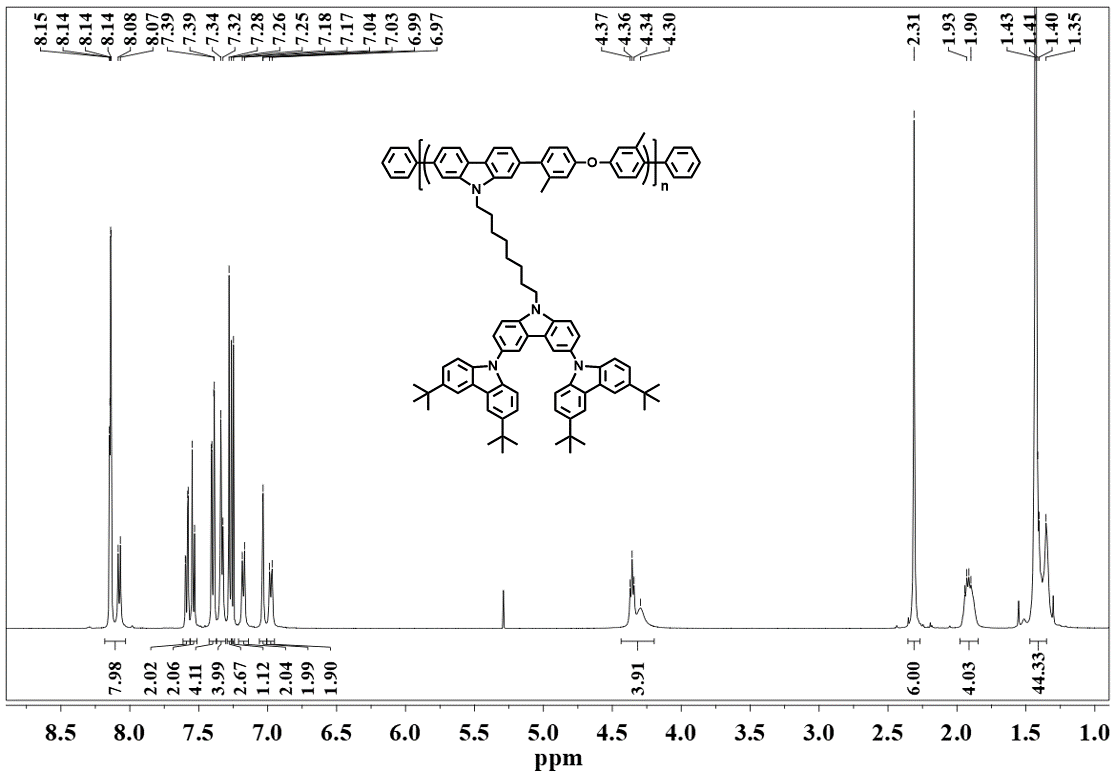


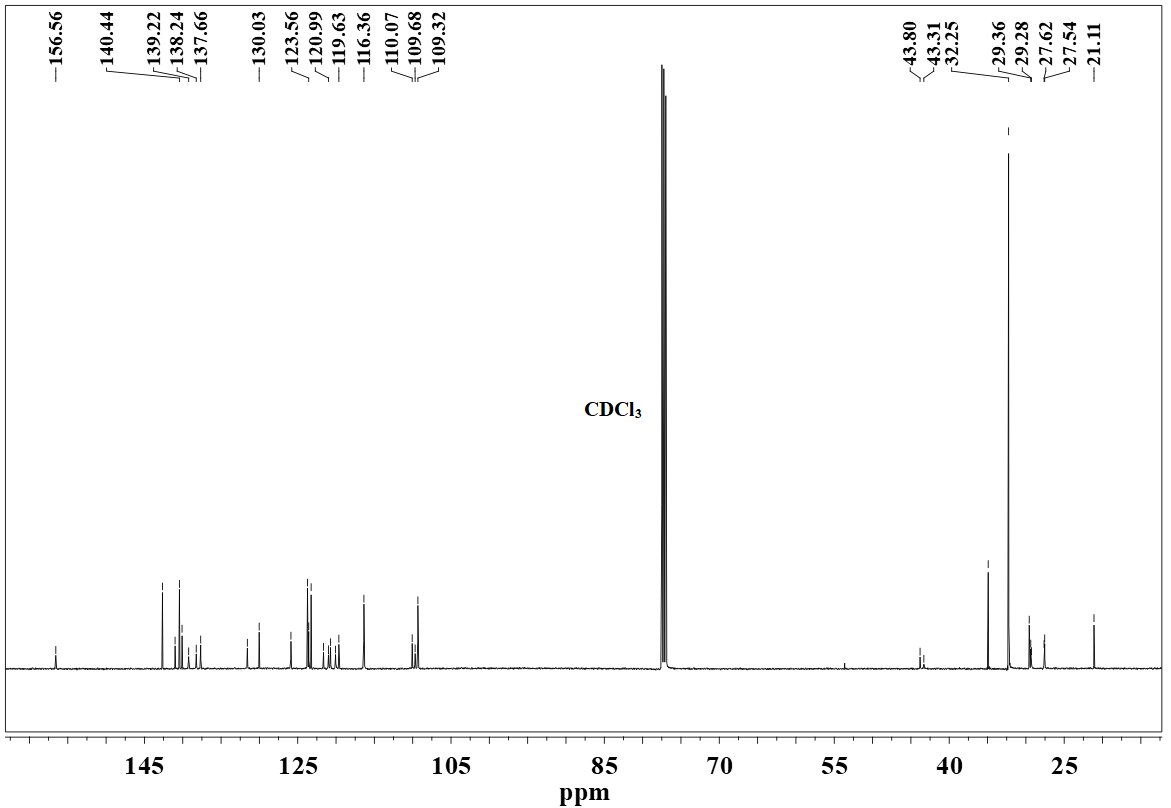


**Figure S9.** ^1^H NMR and ^13^C NMR of polymer D2-PCzDMPE.


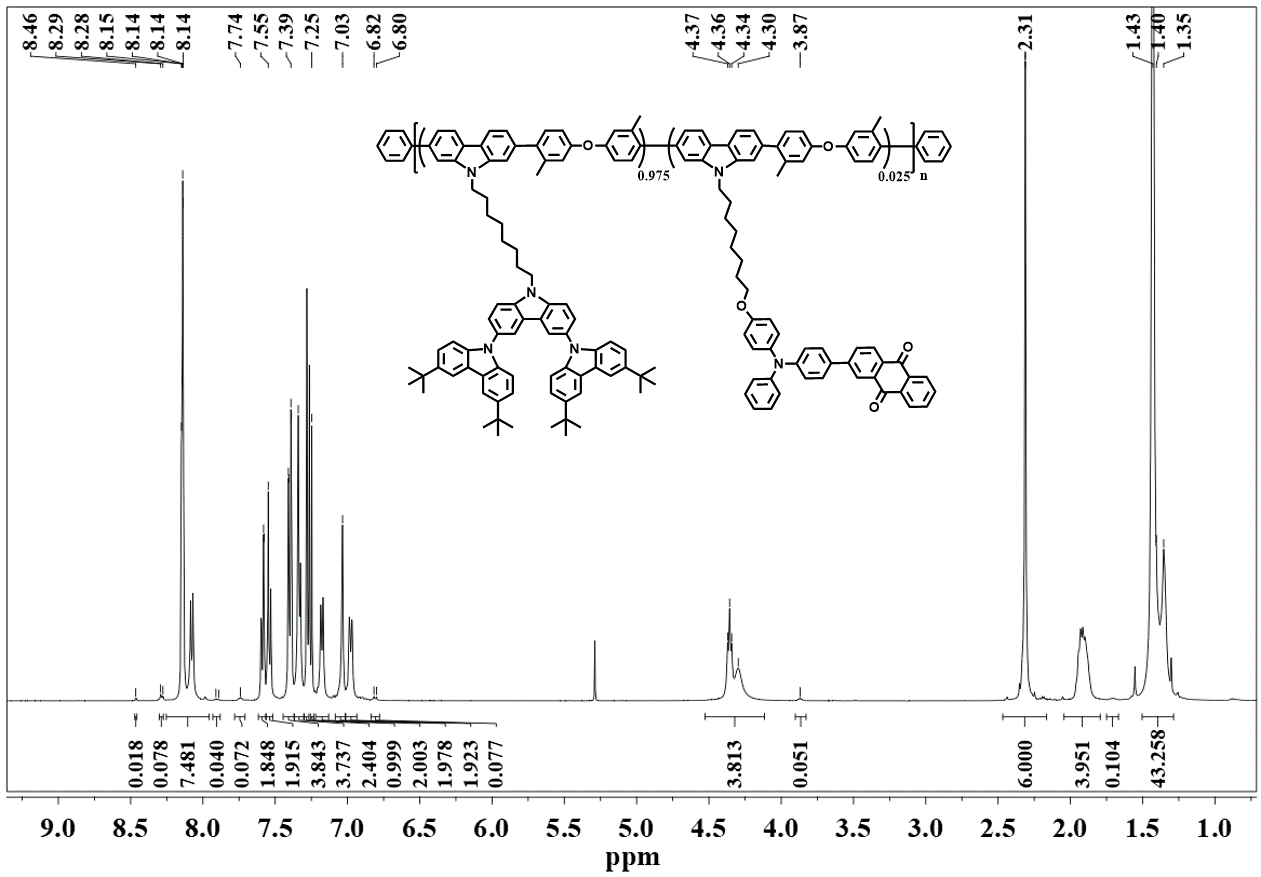


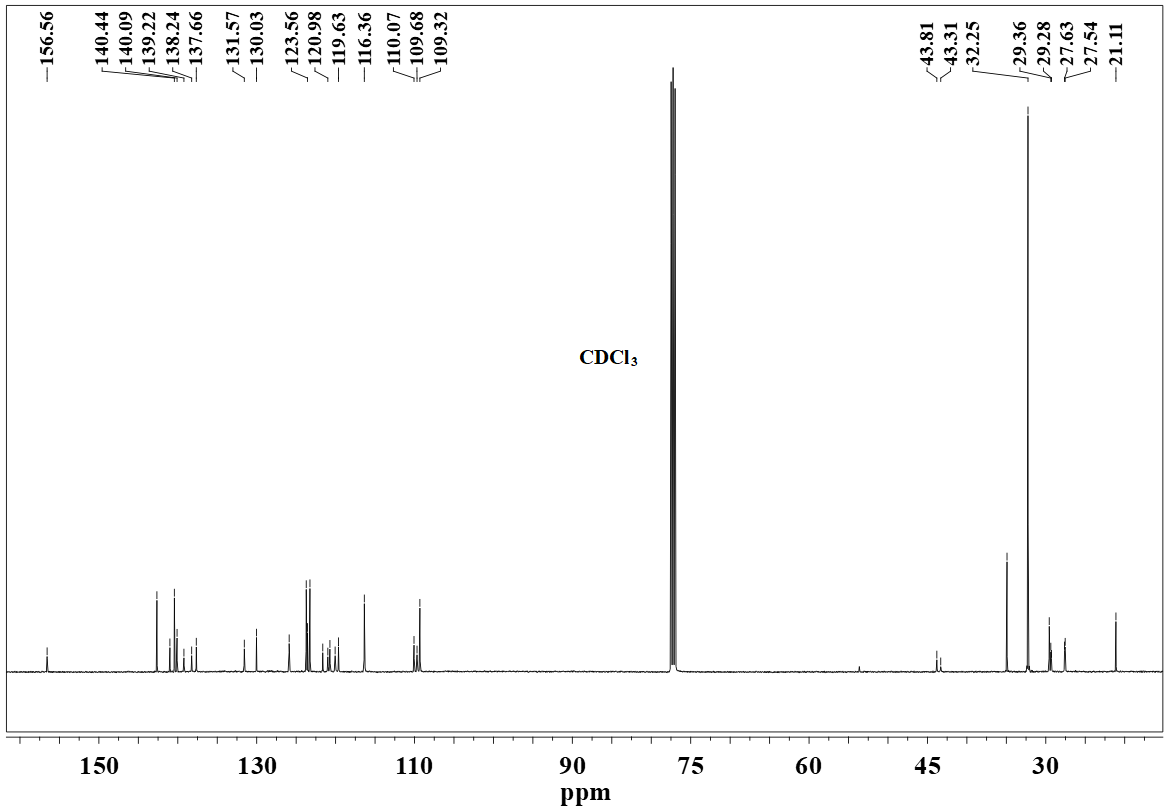


**Figure S10.** ^1^H NMR and ^13^C NMR of polymer D2-PCzDMPE-R2.5.


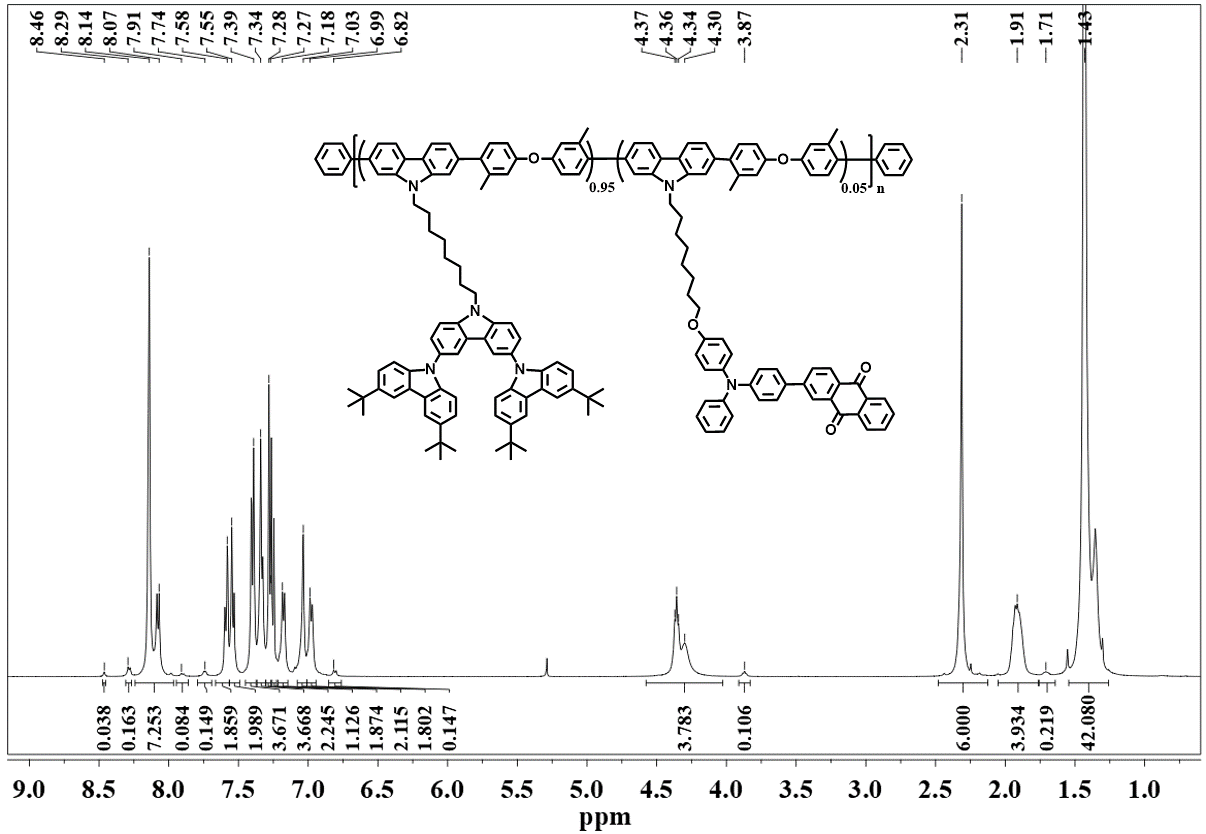


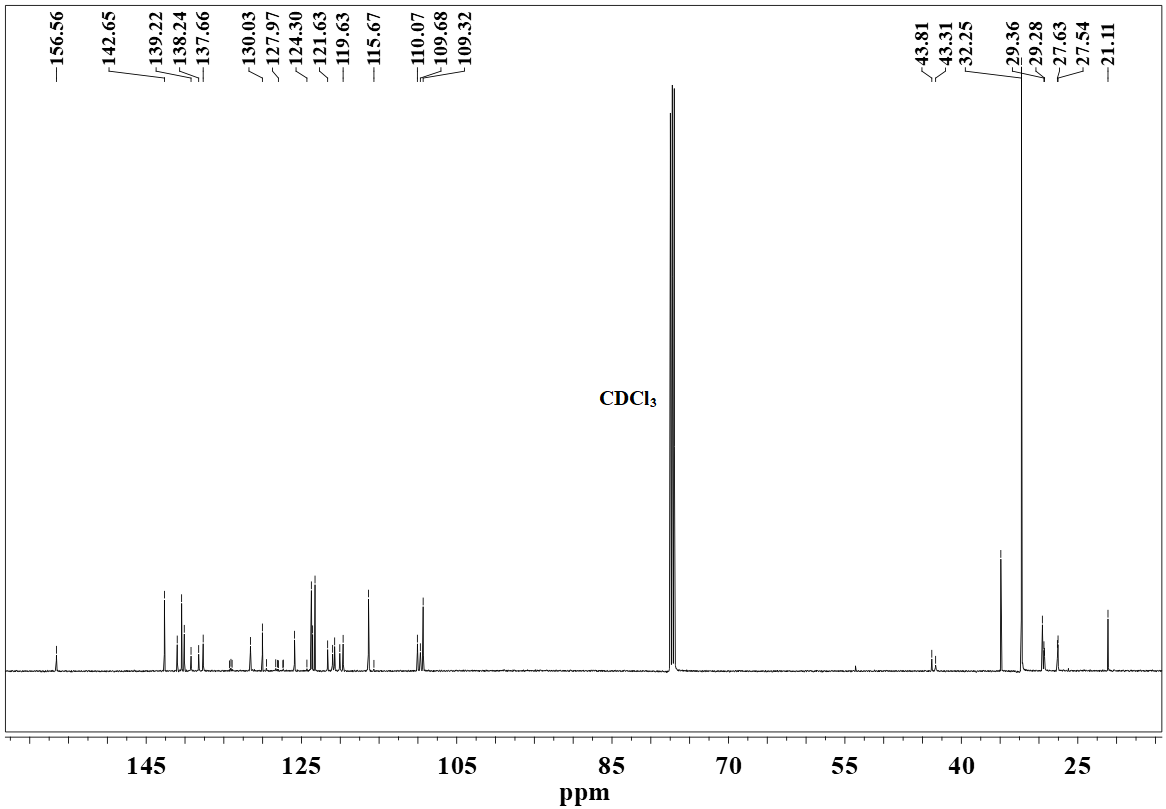


**Figure S11.** ^1^H NMR and ^13^C NMR of polymer D2-PCzDMPE-R5.0.


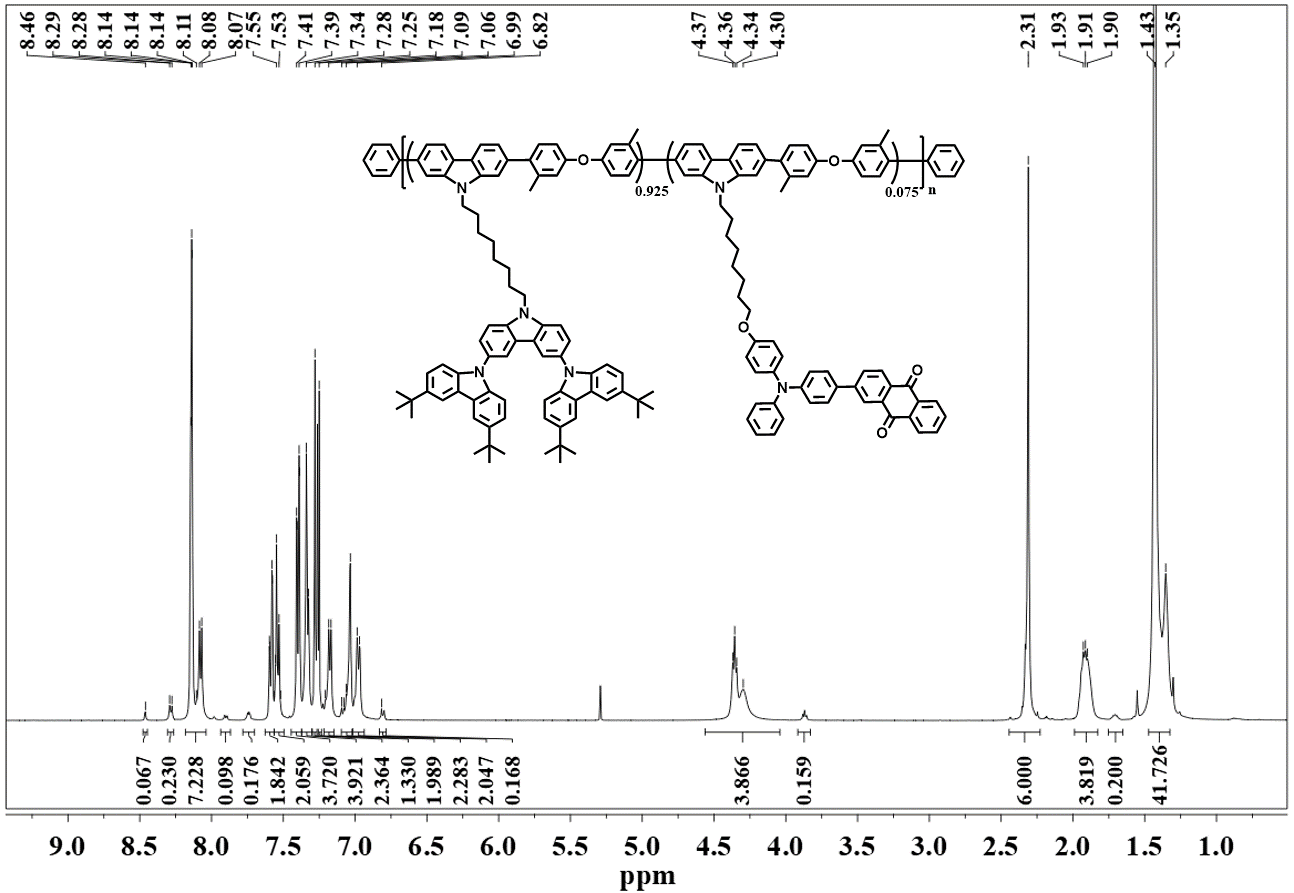


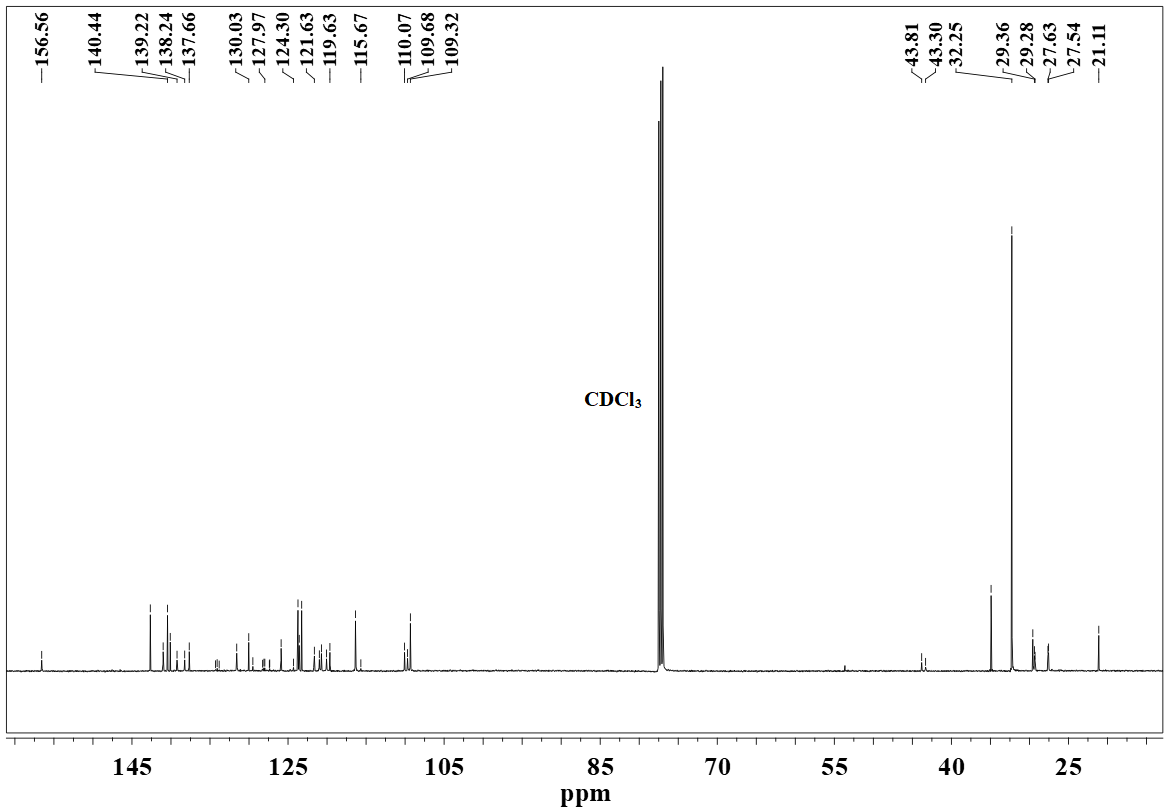


**Figure S12.** ^1^H NMR and ^13^C NMR of polymer D2-PCzDMPE-R7.5.


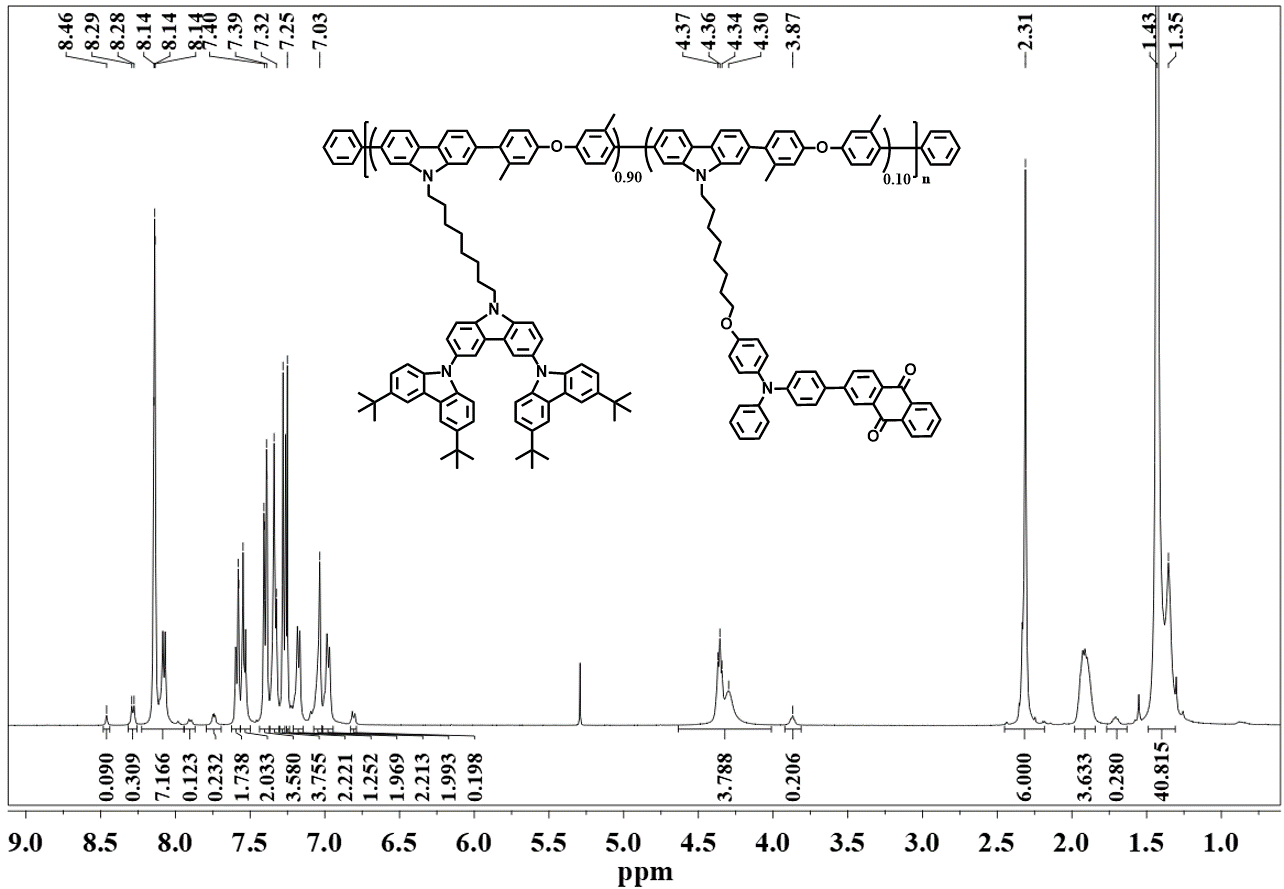


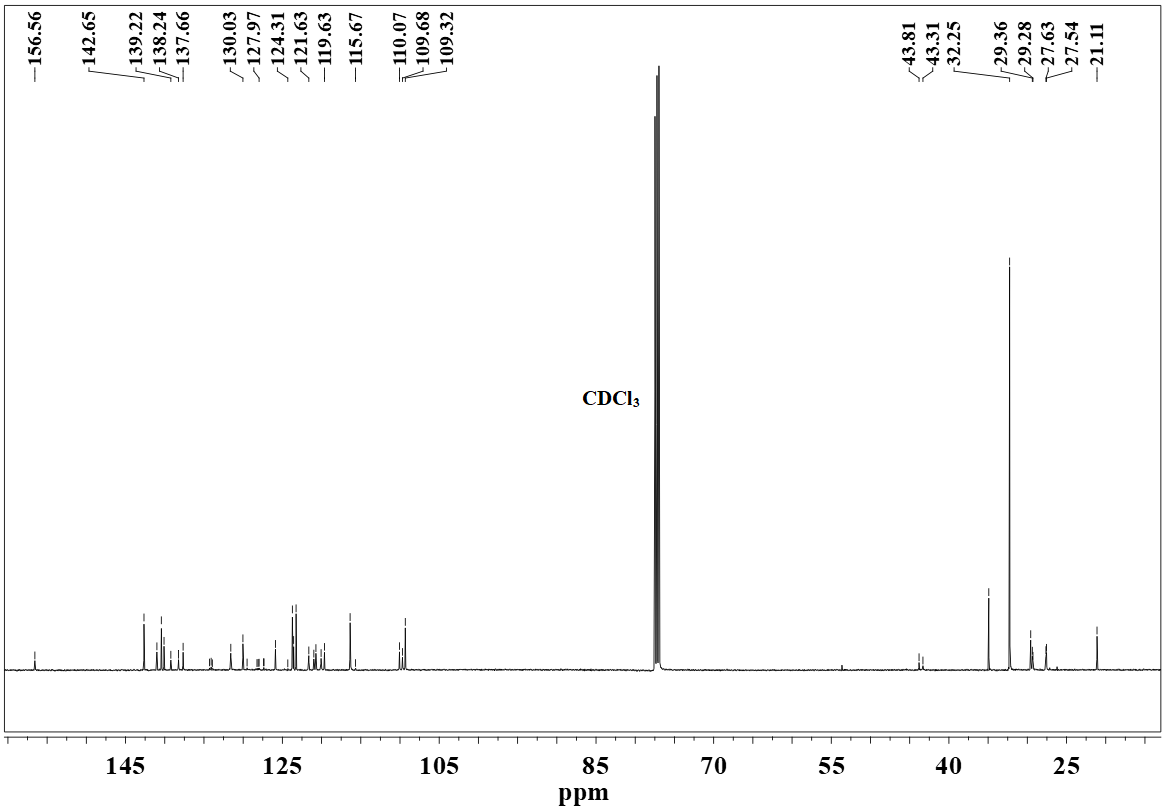


**Figure S13.** ^1^H NMR and ^13^C NMR of polymer D2-PCzDMPE-R10.
